# Interplay between Misplaced Müllerian-Derived Stem Cells and Peritoneal Immune Dysregulation in the Pathogenesis of Endometriosis

**DOI:** 10.1155/2013/527041

**Published:** 2013-06-13

**Authors:** Antonio Simone Laganà, Emanuele Sturlese, Giovanni Retto, Vincenza Sofo, Onofrio Triolo

**Affiliations:** ^1^Department of Pediatric, Gynecological, Microbiological and Biomedical Sciences, University of Messina, Via C. Valeria 1, 98125 Messina, Italy; ^2^Department of Environmental Sciences, Safety, Territory, Food and Health, University of Messina, Via C. Valeria 1, 98125 Messina, Italy

## Abstract

In the genetic regulation of Müllerian structures development, a key role is played by Hoxa and Wnt clusters, because they lead the transcription of different genes according to the different phases of the organogenesis, addressing correctly cell-to-cell interactions, allowing, finally, the physiologic morphogenesis. Accumulating evidence is suggesting that dysregulation of Wnt and/or Hox genes may affect cell migration during organogenesis and differentiation of Müllerian structures of the female reproductive tract, with possible dislocation and dissemination of primordial endometrial stem cells in ectopic regions, which have high plasticity to differentiation. We hypothesize that during postpubertal age, under the influence of different stimuli, these misplaced and quiescent ectopic endometrial cells could acquire new phenotype, biological functions, and immunogenicity. So, these kinds of cells may differentiate, specializing in epithelium, glands, and stroma to form a functional ectopic endometrial tissue. This may provoke a breakdown in the peritoneal cavity homeostasis, with the consequent processes of immune alteration, documented by peripheral mononuclear cells recruitment and secretion of inflammatory cytokines in early phases and of angiogenic and fibrogenic cytokines in the late stages of the disease.

## 1. Introduction

Endometriosis is an estrogen-dependent disease [1] characterized by the ectopic presence and growth of functional endometrial tissue, glands, and stroma, outside the uterine cavity [2, 3]. It affects deeply and negatively woman's quality of life, contributing not only to suffering but also to marital and family problems, to problems related to the achievements of work tasks, and overall to disability in woman's role in modern society [4–7]. Its treatment, medical or surgical depending on each case, on the contrary, could improve and partially restore women's health-related quality of life (HRQoL), like is reported by Jia et al. [8] and Gao et al. [9]. Risk factors for this disease are nulliparity, high education level, and social class (probably because these patients undergo accurate medical controls more easily) [10], although it is widespread across countries and ethnicities, and women continue to experience diagnostic delays in primary care [6]. As is suggested by many authors [11, 12] the risk of endometriosis appears to increase for reproductive health factors that may relate to increased exposure to menstruation (i.e., shorter cycle length, longer duration of flow, or reduced parity). The risk appears to decrease for personal habits that may relate to decreased estrogen levels (i.e., smoking, exercise). Approximately 10% of women in reproductive age are estimated to be affected by this disease [13, 14] and its symptoms, which include acute or chronic pelvic pain (CPP) and abnormal bleeding [12]. Pelvic pain could be expressed as dysmenorrhea, dyspareunia, dysuria, dyschezia, and nonmenstrual chronic pelvic—abdominal muscle pain [15]. Dysmenorrhea is independent of the macroscopic type of the lesions or their anatomical locations and may be related to recurrent cyclic microbleeding in the implants [16]. The severity of dysmenorrhea seems to be significantly correlated with the presence and extent of pelvic adhesions, whereas the severity of CPP and deep dyspareunia is correlated with deep endometriosis on the uterosacral ligaments and extent of pelvic adhesions [17]. For example, Vercellini et al. [18] analysing 1054 consecutive women with endometriosis undergoing first-line surgery found first of all a significant inverse relationship between age at surgery and moderate-to-severe dysmenorrhoea, dyspareunia, and nonmenstrual pain. Moreover, they reported a strong association between posterior cul-de-sac lesions and pain at intercourse. Similar finding was found by Arruda et al. [19] in a smaller cohort study of Brazilian women, in which endometriosis symptoms (especially CPP) were more severe in young women with delayed diagnosis. There is evidence [20] that the typical endometriosis-associated chronic pelvic pain and sensitivity to estrogen could depend, at least in part, by the growth into the ectopic endometrial tissue of a nerve supply. Affected women are at higher risk than the general female population of developing ovarian cancer, and they also may be at increased risk of breast and other cancers as well as autoimmune and atopic disorders [1]. The disease most often affects the ovaries (up to 88% of all cases), uterine ligaments, fallopian tubes, rectum, cervical-vaginal region, and urinary tract. Urinary tract involvement is rare accounting for around 1-2% of all cases [21, 22], of which 84% are found in the bladder [23]. However, endometriosis can be encountered in other abdominal organs such as the liver, pancreas, intestinal tract, spleen [24], gallbladder [25], the abdominal wall, and even the navel [26]. Endometriosis is classified depending on the number, size, and superficial and/or deep location of endometrial implants, plaques, endometriomas, and/or adhesions, as follows: stage I (minimal, 1–5 points), stage II (mild, 6–15 points), stage III (moderate, 16–40 points), and stage IV (severe, >40 points), following the revised American Society for Reproductive Medicine classification for Endometriosis (American Society for Reproductive Medicine, 1996) [27].

## 2. Immune Disturbance of the Peritoneal Microenvironment

Immune system seems to play a key role in the pathogenesis of endometriosis. In these patients, immune alterations occur in the PF (PF) and peripheral blood, in part comparable to those proper of autoimmune diseases. It is widely reported an increase in the number but not in the function of macrophages, abnormalities in the functions and numbers of T and B lymphocytes, a reduction in number and activity of natural killer cells, apoptosis impairment, changes of cytokines and other soluble products in the peritoneal microenvironment.

### 2.1. Macrophages

Macrophages are “master regulators of the innate response to injured, infected, and neoplastic tissues” [28]. The microenvironment may drive the macrophage plasticity toward a transient and reversible polarization. These polar phenotypes are not expressed together, but the activation state of tissue macrophages can change over time. Then, they may be divided into two main populations: the “classically activated” macrophages, named M1 and stimulated by IFN*γ* and LPS, and “alternatively activated” M2 macrophages, stimulated by IL4, IL13, IL10, and TGF*β* [29]. M1- and M2-activated macrophages perform different functions by producing pro- or anti-inflammatory factors. M1 macrophages play endocytic functions via the production of cytokines such as IL1*α*, IL6, IL12, and TNF*α* and reactive oxygen (ROS) and nitric oxide (NO) species [30]. In contrast, M2 macrophages are involved in resolution of inflammation and promotion of tissue repair, by secreting anti-inflammatory and immunosuppressive cytokines, IL10 and TGF*β*, proangiogenic factors, such as coagulation factor XIII and vascular endothelial growth factor (VEGF) associated with a high degree of vascularization in vivo [29]. Resident peritoneal macrophages and peripheral monocytes, recruited from the blood into the peritoneal cavity, physiologically play a pivotal role in the scavenging mechanisms [31–35]. The immune surveillance of the peritoneal microenvironment would be able to prevent ectopic endometrial cells from becoming established. Macrophages are physiologically recruited in injured tissues, where they activate the neo-angiogenic switch, sustain resistance to apoptotic stimuli, and stimulate the proliferation and invasion of precursor cells in order to prompt tissue regeneration. Macrophages recruited in the endometriotic lesions activate a similar program. Several authors indicate that infiltrating macrophages in the endometriotic lesions are activated by signals generated within the same lesions [36, 37] or possibly by the lack of hormone-dependent anti-inflammatory signals in the ectopic but none in the eutopic endometrium [38].

Once endometriosis is established, the cyclic death of endometrial cells, due to progesterone withdrawal, leads to the release of cell debris, erythrocytes, and heme-bound iron in the peritoneal cavity. Recruited macrophages perceive ongoing cell death and tissue damage; in endometriotic patients they activate a reparative/regenerative/angiogenic program that is required for lesion maintenance, growth, and spreading [28]. The persistence of cells dying as a result of progesterone withdrawal within endometriotic lesions could cyclically activate infiltrating macrophages, thus sustaining the inflammation associated to the disease. Chuang et al. [39] reported an apparent impairment in the macrophage ability to phagocytose these cells dying, probably due to the defective expression and function of the class B scavenger receptor CD36 [40]. However, it is difficult to verify whether such defect is cause or consequence of the persistent inflammation of the peritoneal cavity associated to the disease. The pathogenesis of endometriosis results therefore by combination of inappropriate or persistent polarization, leading to tissue damage (increased M1 response) and immune dysfunction (increased M2 response). This allows for persistence of ectopic endometrial tissue. The disease is associated with significant alterations in the number of tissue macrophages expressing M1 or M2 surface markers. M2 activation leads to stimulation of anti-inflammatory cytokine production and inhibition of proinflammatory cytokine expression, thus reducing inflammation. Macrophages may act to suppress the immune response to endometriosis and provide an environment permissive to the growth and progression of endometriosis lesions. It was demonstrated that recruited macrophages largely develop an immunosuppressive phenotype M2, thereby supporting endometriotic cell survival, attachment, and invasion through matrix remodeling, angiogenesis, and lesion maintenance. The reduced phagocytic ability of peritoneal macrophages of women with endometriosis is driven by soluble factors of tissue microenvironment, that determine the macrophage phenotype and function [29]. Then, the recruitment of macrophages into the lesions represents not only an early event in the disease development but also a necessary step for the successful establishment of endometriotic lesions [28]. Macrophages from endometriotic patients and mice with implanted endometriotic lesions (but not peritoneal macrophages from human or murine controls) express typical markers of alternative activation, in particular high levels of scavenger receptors, CD206 and CD163 [41]. CD206, a PRR which belongs to the C-type lectin superfamily, contributes to remove or inactivate inflammatory signals. CD163 mediates endocytosis of haptoglobin-hemoglobin complexes, with degradation of heme-iron components that can be recycled for erythropoiesis [42]. Macrophage polarization results in differential iron management in both humans and mice, with classically activated M1 macrophages that are characterized by iron sequestration [43] and alternatively activated M2 macrophages that are able to internalize and recycle the metal [44]. It is hypothesized that the polarization of infiltrating macrophages toward M2 phenotype provokes a more effective transfer of the metal to epithelial cells, supporting the growth and the spreading of the lesions. In experimental animals, it is reported that infiltrating M2 strongly enhance the growth of endometriotic lesions, suggesting that this program is important for the natural history of the disease. In contrast, mice injected with M1 macrophages do not develop growing minute lesions [41]. Macrophage migration inhibitory factor (MIF) activates macrophages and may therefore play a role in retaining these cells into the inflammatory sites [45]. It was demonstrated [46] that an increased secretion of MIF by peritoneal macrophages of women with endometriosis and further revealed an increased expression of this factor in eutopic endometrium and initial, active, and vascularized endometriotic lesions. Moreover, either local peritoneal fluid or systemic circulating levels of MIF were found to be higher in women with endometriosis and appeared to depend on the disease's stage and major clinical symptoms (pain and infertility) [47]. Interestingly, according to Seeber et al, the association of MIF with cancer antigen (CA)-125, monocyte chemotactic protein 1 (MCP1) and leptin, can diagnose endometriosis in 48% of patients with a specificity equal to 93% [48]. MIF is now known for being a multifunctional factor with a wide spectrum of effects and cell targets. MIF plays an essential role in tumorigenesis, tissue remodeling, and angiogenesis [49]. Recent data from the literature showed an important role for MIF in cell proliferation, inhibition of apoptosis [50], stimulation of metalloproteinases, and induction of angiogenesis [51]. MIF stimulates COX2 expression in ectopic endometrial cells and elicit proangiogenic and proinflammatory phenotype of macrophages, thereby potentiating their capability to stimulate the host angiogenic response and exacerbate the immunoinflammatory reaction occurring in the implantation site [46]. In addition, it was reported that macrophage migration inhibitory factor (MIF) protein secretion and mRNA expression increase significantly in endometriotic cells in response to estradiol. In turn, MIF reciprocally stimulates aromatase protein and mRNA expression, contributing to elevation in estradiol levels. Consequently, this mechanism may establish a positive feedback loop that contributes to develop and aggravate the disease [52].

### 2.2. T Lymphocytes

Several studies demonstrated that defective T-lymphocyte response to autologous endometrial cells was associated with endometriosis. There is evidence that the lymphocyte proliferative response to autologous endometrial cells was decreased in women with endometriosis. The cytotoxicity of T lymphocytes against autologous endometrial cells was also reduced in women with endometriosis [72]. Another mechanism by which endometriotic cells are able to escape from immune surveillance of cytotoxic T lymphocyte is attributable to FasL expressed by endometriotic cells. FasL induces apoptosis of lymphocytes by binding to its receptor, Fas, expressed on lymphocytes. Therefore, cells that are expressing high FasL may cause apoptosis of surrounding lymphocytes and thereby escape from lymphocytes response. Like we argued in our previous work [73], PF of women with endometriosis may have a potential to induce apoptosis of cytotoxic T lymphocytes, directly or indirectly via stimulating endometriotic cells, and contribute to the survival of endometriosis. Besides cytotoxic T lymphocytes, characterized as CD8^+^ T cells, helper T cells or, namely, CD4^+^ T cells are further diminished in their activity in PF from patients with endometriosis, probably because PF homeostasis breakdown suppresses activation of helper T cells [72]. Probably, IL-10, one of the well-known immunosuppressive cytokines, plays a role in this mechanism: we have already evidenced high level of IL-10 mRNA expression in ovarian endometrioma samples [74, 75], and this cytokine is also associated with decreased activated CD4^+^ T cells in endometriotic PF. Moreover IL-4 and IL-10 were shown to be upregulated in peripheral lymphocytes in women with endometriosis. Increased IL-4 expression is also seen in lymphocytes in endometriotic tissues and in PF. On the other hand, production of IFN-*γ* was reduced in peripheral lymphocytes in endometriosis. Likewise, production of IFN-*γ* in peritoneal cells and IFN-*γ* concentrations in PF were decreased in endometriosis [72]. The PF mononuclear cells (PFMCs), as well as endometriotic cells, secrete different patterns of cytokines [34, 35, 52, 76] which drive the differentiation programs of CD4^+^T cells toward Th1, Th2, and Th17 and Tregs. Th1 cells are characterized by T-bet and STAT1 and 6 and the production of IL-2, IFN-*γ*, and TNF-*α*. Th2 cells are characterized by GATA-3 and STAT5 and 6 and the production of IL-4, IL-5, and IL-13. In very recent years, however, the Th1/Th2 dogma has been challenged by the introduction of two other subsets of T cells: Th17 cells and regulatory T (Treg) cells. Since the eutopic endometrium behaves like an immune regulatory tissue, the specific activities of these immune cells are crucial. Th17 cells are characterized by RORC and produce IL-17A, IL-17F, and IL-22 [77]. Recently, Osuga et al. [72] demonstrated the presence of Th17 cells in PF of endometriosis and further that IL-17 stimulates endometriosis stromal cells proliferation, their IL-8 and cyclooxygensase-2 expression. Tregs are characterized by FOXP3 and produce IL-10 and TGF-*β*, suppressing activation of the immune system and thereby maintaining immune system homeostasis and tolerance to self-antigens [78–82]. A mouse model study [83] reported in late-stage of the disease a switch toward Th2 and Treg cell profiles, with an overrecruitment of Foxp3^+^ CD4^+^ Tregs in the draining lymph nodes. This is congruent with our previous reported results [34]. It was proposed that the preserved Treg cells seen in women with endometriosis decrease the ability of newly recruited immune cell populations to effectively recognize and target endometrial antigens during menstruation, allowing survival and implantation of shed endometrial cells [72]. As proposed by Podgaec et al. [84], the immune cells (macrophages, dendritic cells, NK, CD4^+^, and CD8^+^ lymphocytes) responsible for local surveillance profile could have their activity suppressed by Treg cells, a fact that would prevent the ectopic endometrial cells from being removed from the peritoneal cavity. This, together with endometrial cells decreased apoptosis rate (see after), may cause perpetuation of disease growth. Moreover, it seems that hormonal therapies for endometriosis not only affect directly the endometriotic cells but also alter the immunological environment and thus in turn contribute to the control of endometriosis. For example, GnRH analog therapy was reported to increase total T lymphocytes number in peripheral blood and T-lymphocyte activity in peripheral blood and PF.

### 2.3. B Lymphocytes

B lymphocytes are responsible for humoral immune response, principally producing antibodies against antigens. In the pathogenesis of endometriosis, they have been suggested to play roles by secreting autoantibodies. Focusing on the role of B lymphocytes in the pathogenesis of endometriosis, particularly autoimmune responses, could be elicited via two major types of autoantibody: antibodies that specifically respond to the endometrium and antibodies that are commonly observed in various autoimmune disorders. Autoantibodies that are frequently found in patients with various autoimmune diseases such as antinuclear antibodies, anti-DNA antibodies, and antiphospholipid antibodies have also been observed in women with endometriosis. This suggests that endometriosis is associated with abnormal polyclonal B-cell activation, a classic characteristic of autoimmune disease. The association between autoantibody and endometriosis may also explain endometriosis-related infertility, as these antibodies might bind to not only the endometrium but also embryos and sperms [72]. 

### 2.4. Natural Killer Cells

NK cells destroy target cells by releasing small cytoplasmic granules of proteins that induce apoptosis. A possible link between NK cells and endometriosis was initially arisen from a study which showed that NK cells in peripheral blood have an ability to destroy endometrial cells. The NK activity and the cytotoxicity against autologous endometrial cells were decreased in women with endometriosis and correlated with the severity of the disease. The decreased cytotoxicity to endometrial cells in women with endometriosis is mainly because of a defect in NK activity but is also partially because of a resistance of the endometrium to NK cytotoxicity [85]. Recently, Sikora et al. [86] suggested that endometriosis may be related to a defect of NK cell cytotoxicity function in the ability to eliminate endometrial cells in ectopic sites. Alternations of the innate immunity mediated by NK cells may promote impairments or disrupt functions of adaptive immunity, which can contribute to development and progression of endometriosis and infertility associated with endometriosis. The reduction of cytotoxic activity of NK cells was also observed in PF of endometriotic women. In particular, Oosterlynck et al. [85] found that PF taken from patients with endometriosis had greater suppressive effect on NK cells cytotoxicity compared to PF from healthy women, suggesting the presence of substances which suppress NK cells cytotoxic activity. Impaired NK cell cytotoxic activity may be a primary cause of development of endometriosis, by allowing endometrial cells escape from their attacks. 

### 2.5. Apoptosis Impairment

During endometriosis a breakdown occurs in endometrial and peritoneal homeostasis caused by cytokine induced cell proliferation and dysregulation of apoptosis [34, 87–89]. Execution of the programmed cell death is a process that can be triggered by many apoptotic signals, and it occurs via two main pathways. Both pathways stimulate an intracellular cascade of events that leads to cell death. The intrinsic pathway is initiated from mitochondria, whereas the extrinsic pathway is activated by death ligands (FasL and TNF*α*) on the cellular surface membrane that engage their respective receptors (Fas and TNFR1/TNFR2) on the surface membrane of target cells [90].

#### 2.5.1. Apoptosis Intrinsic Pathway

Intrinsic cell death pathway is regulated by BCL2 family proteins. This family is divided into three different subclasses based on structural and functional features. BCL2 (and its antiapoptotic orthologues) seems to inhibit apoptosis by the preservation of mitochondrial membrane integrity as its hydrophobic carboxyl-terminal domain is linked to the outer membrane. BCL2 prevents BAX/BAK oligomerization, which would otherwise lead to the release of several apoptogenic molecules from the mitochondrion. It is also known that BCL2 binds to and inactivates BAX and other proapoptotic proteins, thereby inhibiting apoptosis [91]. Numerous alterations in the apoptotic intrinsic pathway, including a significant up-regulation of the antiapoptotic molecule Bcl2 and a significant downregulation of the proapoptotic factor Bax, occur in endometriosis [46].

#### 2.5.2. Apoptosis Extrinsic Pathway

*Fas and FasL System. *Fas (DR2/CD95/Apo-1) is a type I cell membrane protein (mFas) with an extracellular domain that binds FasL and a cytoplasmic domain that transduces the death signal [92, 93]. FasL (CD95L/CD178/Apo-1L) is a type II cell membrane protein (mFasL) which is inducibly expressed in lymphocytes and constitutively expressed in cells present in immune-privileged organs [94, 95]. We have previously reported [73] that Fas/FasL system is dysregulated progressively throughout the course of the disease, with the result that endometriotic cells do not undergo Fas/FasL-mediated apoptosis because they do not receive a death signal from PFMCs, thus implanting themselves and surviving outside of the uterus. Paradoxically, endometriotic cells become themselves capable of killing PFMCs, and this may allow their establishment in the peritoneum, which in turn becomes an immune privileged environment [96–99].

*TNF*α* and TNFR1/TNFR2 System. *TNF*α*, belonging to TNF superfamily, is synthesized as a 26 kDa transmembrane type II protein (mTNF*α*). 17 kDa soluble form of the cytokine (sTNF*α*), that retains its biological activity, rises by TACE action on mTNF*α*. Both forms of TNF*α* coexist as mono-, di-, or trimeric proteins. The last form only of both cytokines is biologically active and exerts its effects interacting with two different transmembrane receptors of TNFR superfamily, TNFR1 and TNFR2. TNFR superfamily consists of two main groups of receptors: the first group includes death receptors, characterized by the presence of the death domain (DD) in their intracellular region, whereas the second one does not have DD. TNFR1 is a 55 kDa protein which belongs to the first group of receptors and is expressed in almost all cell types. TNFR2 is a 75 kDa protein of the second group and is expressed only in certain cell types, including T cells. Whereas sTNF*α* binds both receptors but only activates efficiently TNFR1, mTNF*α* can bind and activate both TNFR1 and TNFR2 [100]. Peritoneal microenvironmental changes could depend, at least in part, also by TNF*α* and TNFR1/TNFR2 system. TNF*α*, upon binding TNFR1 or TNFR2, appears to mediate different biological activities ranging from the proliferation, differentiation, and angiogenesis to the activation of apoptosis [101]. We have showed that also this last described system is dysregulated during endometriosis and addresses immune responses according to disease's stage [101]. About this, we must consider that Zhao et al. [102] analyzed 26 single-nucleotide polymorphisms (SNPs) in the coding and the promoter region of the TNF*α* gene in 958 endometriosis cases and 959 controls, and they concluded that TNF*α* gene is not a major susceptibility gene for endometriosis. However, other data provided by another group [103] point out that the frequencies of the TNF*α* T/C/C haplotype allele and the TNFR2 G/G/T haplotype allele are significantly decreased in women with endometriosis compared to women without endometriosis, thus associating these haplotype alleles and polymorphisms to the disease. Moreover, an Indian population study [104] suggests an association between TNF*α*-C850T polymorphism and endometriosis.

### 2.6. Changes of Cytokines and Other Soluble Products in the Peritoneal Microenvironment

Knowledge of these factors is indispensable for the development of strategies for prevention and targeted treatment of endometriosis. Changes of the wide range of soluble products including (a) cytokines, (b) angiogenic factors, (c) adhesion molecules, (d) hormones, (e) prostaglandins (PGs), and (f) reactive oxygen species (ROS) are characteristic findings in the peritoneal microenvironment of endometriotic women [105].

#### 2.6.1. Cytokines

The role of cytokines in the development of endometriosis is emphasized in the literature. Several researchers, in independent works, assessed the levels of cytokines involved in immune response patterns Th1 and Th2 in patients with endometriosis. Podgaec et al. [84] noted an increase in the levels of IFN-gamma and IL-10 in patients with endometriosis, evidencing the coexistence of both responses. However, it a predominance of IL-4 and IL-10 was observed, thus reflecting a polarization toward Th2 immune response [106]. In a previous study, we showed a prevalence of Th1 profile cytokines in the PF of women with endometriosis at minimal and mild stages, whereas Th2 profile cytokines prevailed in severe stages [34]. Particularly, we reported that serum and PF levels of TNF-*α* were very high at the early stage and decreased with the severity of the disease. TGF-*β* levels were high and increased with the severity of the disease, particularly in the PF. Serum and PF IL-8 as well as MCP-1 concentrations at all stages were high, yet showed an opposite behaviour in both biological fluids. In fact, IL-8 and MCP-1 serum levels were higher at early stages and decreased with the severity of the disease, whereas the PF levels increased with the worsening of the disease [34]. 

*IL1*. Data from the literature evidenced an impairment of the secretion of the IL-1 cytokine family in endometriosis. For example, a marked imbalance was reported between IL-1 and its natural inhibitor IL-1 receptor type 2 (IL1R2). This points to a deficiency in the local control of IL-1 that, in view of the cytokine's elevated levels and potent proinflammatory, angiogenic, and growth-promoting effects, may contribute to endometriosis development. sIL1R2 significantly downregulated the expression of major cell adhesion receptors (*α*v and *β*3 integrins), matrix metalloproteinases (MMP-2 and -9), and VEGF [107]. Moreover, invalid IL-1*β* and IL-18 maturation by interleukin-1 converting enzyme (ICE) may be an important pathogenic factor in endometriosis [108].

*IL6 and IL8*. Concentrations of these cytokines are elevated in the PF of endometriotic patients. It was suggested that the inflammatory process, typical of the disease, once started continues being activated constantly, perpetuating itself via high concentrations of IL-6 [109].

Significantly higher IL-6 and IL-10 levels were found in moderate-to-severe but not in minimal-to-mild endometriosis as compared to controls [110]. Moreover, serum levels of both IL-6 and IL-8 are significantly higher in patients with ovarian endometrioma, but not in the presence of deep infiltrating endometriosis [111].

*IL10*. Studies regarding this cytokine reported conflicting results. Some authors share the evidence that no significant differences were observed in the PF of endometriotic women in respect to controls, whereas others showed an elevation [112]. Contradiction can be explained by the velocity of production and consumption of inflammatory products, making comparisons difficult [84]. In particular, according to these latter authors, increased IL10 production may partially contribute to the disturbed immune regulation in patients with endometriosis, because it attenuates TNF-*α*-induced IL6 synthesis via NF-kappaB and MAPK pathways in endometriotic cells. 

*IL15*. The levels of this cytokine are increased in PF of women with endometriosis. However, these levels are inversely correlated with the depth of invasion and disease stage, suggesting a possible role for IL15 in the early pathogenesis of endometriosis [113].

*IL17*. Zhang et al. [114] related an elevation of IL17 levels in the PF of patients with minimal or mild endometriosis stages. This relation was even more positive when endometriosis at these stages was associated with infertility. Conversely, Others did not find any difference in IL17 peritoneal fluid concentration in patients with or without endometriosis.

*IL18*. PF levels of this cytokine are elevated in women with peritoneal, minimal-to mild-stage endometriosis [115]. Moreover, the increased concentrations of IL-18 in PF of endometriotic women do not correlate with menstrual cycle phase [109].

*IL19 and IL22*. Serum levels of these cytokines are decreased in women with ovarian endometrioma [116].

*IL33*. Peritoneal as well as serum levels of IL33 are elevated in women with endometriosis and principally in deeply infiltrating endometriosis. Elevated serum but not peritoneal IL33 levels are correlated with the intensity of preoperative painful symptoms and with the extent and severity of the deeply infiltrating endometriosis [117].

*TNF*α**. A large body of evidence indicates that TNF*α* and IL1*β*, typical inflammatory cytokines, are involved in macrophage activation, inflammatory change, and enhanced angiogenesis to develop endometriosis [118]. A pivotal role of TNF*α* in endometriosis is corroborated by the finding that TNF-*α*-targeted suppression by specific drugs inhibits the development of endometriosis in baboons [119, 120]; TNF*α* has also been shown to be elevated not only in the peritoneal fluid but also in the serum of women with the disease. Indeed, there is a positive correlation between peritoneal levels of TNF*α* and the size and number of active lesions [121]. In addition to its proinflammatory functions, TNF*α* also stimulates the expression of matrix metalloproteinases in endometrial tissue [122]. Matrix metalloproteinases are known to play a role in tissue remodeling and invasion of endometriotic lesions [123]. These data suggest that TNF*α* may influence the establishment and progression of disease and that an antagonist of TNF*α* may be effective in treating patients with endometriosis.

*TGF*β**. There is evidence that concentrations of this cytokine are ten times higher in peritoneal fluid of patients with endometriosis compared to those without the disease. In the inflammatory process, high levels of TGF*β* may occur in the regeneration process, inducing adhesion formation and the appearance of fibrotic tissue and stimulating Treg cells that are elevated to regulate the exacerbated immune response [84]. However, Meta-analyses of the available data showed that the association between TGF-*β*1-509C/T polymorphism and susceptibility of endometriosis was not significant [114].

#### 2.6.2. Angiogenic Factors

The ability of ectopic endometrial cells to invade the underlying basement membrane represents a further necessary step for lesions establishment. Endometrial tissue invades even intact serosal membranes, indicating that a previously disrupted peritoneum is not a requirement. Invasion is a prerequisite for the organization of the ectopic endometrial cells in tridimensional cysts but is not sufficient: novel vessels are also necessary. Angiogenesis may play an important role in the pathogenesis of endometriosis. Endometriotic implants require neovascularization to proliferate, invade the extracellular matrix, and establish an endometriotic lesion, similar to tumour metastases. Several studies have reported, in endometriosis, an increase in levels of VEGF-A, angiogenic factor playing a major role in the progression of the disease. Thrombospondin-1 (TSP-1), an inhibitor of angiogenesis, may also be involved in endometriosis, in which vessel formation occurs. Moreover, the same authors observed an increase in VEGF-A levels and proteolytic factors, like urokinase plasminogen activator (uPA) and metalloproteinase-3 (MMP-3), in peritoneal fluid from patients with endometriosis in comparison with women without the disease. These factors may enhance the angiogenic and proteolytic capability of ectopic tissue to facilitate the implantation process [28]. MicroRNAs (miRNAs) may be the main regulators of angiogenesis. Several studies [124–129] have indicated that endometrium and PF from women with endometriosis have different expression patterns of several angiogenic and proteolytic components in comparison with endometrium and peritoneal fluid from control women, suggesting that these systems play a role in the pathogenesis of endometriosis. Braza-Boïls et al. [130] evaluated the influence of PF from women with or without endometriosis on the expression of six miRNAs that modulate angiogenesis, as well as several angiogenic and proteolytic factors, in endometriotic and endometrial cell cultures from women with and without endometriosis. All of these alterations could dysregulate miRNA expression in stromal cells of endometrial fragments migrated to peritoneum, facilitating the implantation of ectopic lesions. The study elucidates that peritoneal fluid from women with endometriosis induces the highest decrease in angiogenesis-related miRNAs and the highest increase in VEGF-A protein levels in endometrial cell cultures from patients. In conclusion, this “in vitro” study indicates that peritoneal fluid from women with endometriosis modulates the expression of miRNAs that could contribute to the angiogenic and proteolytic disequilibrium observed in this disease. Another evidence suggests that increased levels of VEGF-A may be associated with a decreased rate of pelvic adhesion formation in the course of endometriosis [131]. Regarding to angiogenic factor polymorphisms analysis, Cosín et al. [132] suggested that the VEGF 936C/T polymorphism may be associated with the risk of endometriosis. Moreover, they showed that endometrium and PF from women with endometriosis showed an increase in VEGF levels. Other data [133] seem to suggest that an increased frequency of the +405CC polymorphism in VEGF was observed in the patients with endometriosis as compared with the controls.

#### 2.6.3. Enzymes and Adhesion Molecules

The establishment of endometriotic lesions in the peritoneal cavity requires adhesion, migration, invasion, and proliferation of the ectopic endometrial tissue [134]. For example, the expression levels of MMP-2 and MMP-9 were higher in women with endometriosis [135]. In fact, the enzymes play important roles in the ectopic adhesion, invasion and implantation, and neovascularisation of the endometrium. MMP-2 and MMP-9, by degrading extracellular cellular matrix and promoting the release of key factors, play a critical role in the pathogenesis of endometriosis [129]. In addition, MMP-2 and MMP-9 are elevated in the urine of patients with endometriosis compared to control. An immunohistochemical study revealed that MMP-9 expression is higher in endometriosis than proliferative endometrium [136].

A substantial body of evidence suggests that a large number of mediators, including cell adhesion molecules such as intercellular adhesion molecule-1 (ICAM-1, CD54) and vascular cell adhesion molecule-1 (VCAM-1, CD106) [137] as well as proinflammatory cytokines such as TNF-*α*, IL1, IL6, and IL8, and chemokines such as MCP-1, play key roles in the pathogenesis of endometriosis. These factors are present in PF as well as in endometriotic implants, and TNF-*α* in particular is regarded as a critical regulatory molecule in eliciting inflammatory immune responses in endometriosis [138]. Interestingly, ICAM-1 is expressed not only in endometrial stromal cells in human endometrium in situ but is also markedly expressed on cultured human endometrial stromal cells [139]. Human ectopic endometrial stromal cells strongly express ICAM-1, and these cells express a higher level of ICAM-1 than eutopic endometrial stromal cells in patients with endometriosis [140]. These data suggest that there may be cross-talk between endometrial stromal cells and leukocytes in normal as well as in endometriotic endometrium and peritoneal fluid via ICAM-1 and its receptors. Furthermore, significantly increased ICAM-1 expression in ectopic endometrial stromal cells from endometriomas is enhanced by stimulation with proinflammatory cytokines such as IL-1*β* and IFN-*γ*, indicating the important role of peritoneal cytokine milieu in the regulation of ICAM-1 expression in endometriotic stromal cells and consequently in the pathogenesis of endometriosis [137]. In addition to ICAM-1, VCAM-1 is also expressed in human endometrial stromal cells. It was observed that TNF*α* markedly augments the expression of ICAM-1 and VCAM-1 [140]. ICAM-1 is involved in the impairment of NK cell function in endometriosis. In patients with endometriosis, elevated expression of ICAM-1 on the human endometrial stromal cells gives rise to the shedding of soluble ICAM-1 (sICAM-1) from the cell surface into the peritoneal cavity, where sICAM-1 binds to the cell membranes of NK cells and interferes with their cytotoxic function, consequently hampering NK cell-mediated removal of ectopic endometrial cells in the peritoneal cavity, and can lead to the development of endometriosis. Moreover, it was found that endometrial release of sICAM-1 is directly correlated with the number and score of endometriotic implants [141]. During menstrual phase, increased endometrial mRNA levels of *α*V integrin, combined *α*V*β*3 integrins were observed in women with endometriosis. Women with endometriosis had increased peritoneal mRNA expression of VCAM-1 during menstrual compared with luteal phase [142].

#### 2.6.4. PGs

Data from women with endometriosis and a murine model of the disease showed that expression of annexin A2 in peritoneal macrophages is inhibited by prostaglandin E2 (PGE2), and this impairs the phagocytic ability of macrophages. The level of annexin A2 mRNA in the macrophages was reduced by PGE2 via the EP2/EP4 receptor-dependent signaling pathway [143]. Endometrial PGE2 and PGF2*α* act as potent vasoconstrictor on the spiral arterioles. PG production, spiral arteriole vasoconstriction, and local hypoxia in turn regulate the production of chemokines, such as IL-8 (CXCL8) and CXC chemokine ligand 12 (CXCL12) stromal cell-derived factor (SDF-1) [28]. Increased PG concentrations in the PF of infertile women with endometriosis have been reported. COX-2 expression was upregulated in endometriotic tissue. Well known as a potent vasodilator, PGE2 may play a role in endometriosis-associated angiogenesis and further contribute to ectopic endometrial cell growth. Moreover, PGE2 appeared to stimulate the expression of aromatase, an essential enzyme in estrogen synthesis in ectopic endometrium, which may favor the ectopic implantation and growth of endometrial tissue. Aromatase, the rate-limiting enzyme for the synthesis of estrogen, is aberrantly expressed in endometriotic implants. Aberrant expression of COX-2 and PGE2 secretion by ectopic endometriotic implants has been reported, although the underlying mechanism is not clearly understood [49]. Epigenetic changes favoring the transcription of the aromatase gene in the endometrium allow endometrial cells to survive in ectopic locations by producing estrogens that spare them from destruction through activated macrophages. Local estrogen production hastens prostaglandin synthesis by stimulating COX-2 activity, thus creating a self-perpetuating sequence of augmented estrogen formation and enhanced inflammation. Repetitive retrograde menstruation reintroduces aromatase-positive endometrial cells endowed with the capacity to implant and invade the peritoneum [144].

#### 2.6.5. Hormones

The biologically active estrogen estradiol (E2) is the best-defined mitogen for the growth and inflammation processes in the ectopic endometriotic tissue. Progesterone and progestins were used in therapy to limit growth and inflammation in endometriosis, but a portion of patients do not respond to treatment with progestins. Bulun et al. [145] reported that this is indicative of a resistance to progesterone action, related to an overall reduction in the levels of progesterone receptors (PRs) and the lack of the PR isoform named progesterone receptor B (PR-B). In normal endometrium, progesterone acts on stromal cells to induce secretion of paracrine factor(s). These unknown factor(s) act on neighboring epithelial cells to induce the expression of the enzyme 17beta-hydroxysteroid dehydrogenase type 2 (17beta-HSD-2), which metabolizes the biologically active estrogen E2 to estrone (E1). In endometriotic tissue, progesterone does not induce epithelial 17beta-HSD-2 expression due to a defect in stromal cells. The inability of endometriotic stromal cells to produce progesterone-induced paracrine factors that stimulate 17beta-HSD-2 may be due to the lack of PR-B and very low levels of progesterone receptor A (PR-A) observed in vivo in endometriotic tissue. The end result is deficient metabolism of E2 in endometriosis giving rise to high local concentrations of this local mitogen [146]. Changes in estradiol homeostasis have been locally observed in endometriosis. A balance was observed between local 2-methoxyestradiol production and angiogenesis, which could promote the development of endometriotic lesions [147].

A local increase in estrogens levels is characteristic of patients with ovarian endometrioma, and this condition could promote endometriotic cell proliferation [148–150]. Moreover, Pabona et al. [151] investigated the expression of Krüppel-like factor 9 (KLF9), a progesterone receptor-interacting protein, in eutopic endometrium of women with and without endometriosis. They reported that the loss of coregulation by KLF9 on WNT-signalling component expression, in human endometrial stromal cells, may account for progesterone resistance in endometriosis. According to Vinatier et al. [152], these changes may influence the development of the peritoneal surface metaplasia or of Müllerian residues.

#### 2.6.6. ROS

17*β*-estradiol (E2) is known to play important roles in the processes that control cell division, differentiation, and proliferation and is considered a major risk factor in the development and progression of endometriosis. It was reported that H_2_O_2_ is a signaling molecule that downregulates apoptosis in endometrial cells, supporting the fact that endometriosis, albeit a benign disease, shares some features with cancer such as decreased catalase levels [153].

## 3. Genetics

Many aspects of female reproductive function are strongly influenced by genetic factors, and numerous studies have attempted to identify susceptibility genes for endometriosis. Family studies of endometriosis indicate that close relatives of patients with endometriosis have an increased risk for the disease [154], suggesting that genetic components perhaps contribute to endometriosis. Recently, several lines of genetic-association studies have revealed associations between the development of endometriosis and certain genetic polymorphisms, although the genes that play a role in susceptibility to the development and progression of endometriosis are unknown [155]. It is widely accepted also that this disease has a family tendency, suggesting an important role of genetic factor in the pathogenesis: for example, Kashima et al. [156] analyzing 339 patients with endometriosis detected sisters with the same disease in 8.8% of cases. Similar finding are reported by Kennedy et al. [157]. Others [158] performed an extensive review of studies of association between genetic variation at common DNA polymorphisms and variation in disease susceptibility and reported that over 600 positive associations have been reported, including single nucleotide polymorphisms (SNPs). This, remembering also that is very important the to take into account gene-environment interactions with known epidemiologic risk factors [159–161]. Among all SNPs, Falconer et al. [162] proposed that genetic polymorphism of hormone receptors, growth factor, and human leukocyte antigen system components showed a relatively stronger correlation than the others. To better understand endometriosis-related genomic regions, Treloar et al. [53] conducted a linkage study of 1,176 families (931 from an Australian group and 245 from a UK group), each with at least two members with surgically diagnosed disease, and identified a region of significant linkage on chromosome 10q26 and another region of suggestive linkage on chromosome 20p13. Considering that endometriosis is clearly an estrogen-dependent disease, there are many reports of positive associations with numerous polymorphisms involving sex steroids production and metabolism: for example, a consistent work by Singh et al. [54] was conducted on eutopic and ectopic (ovarian) endometrium from patients with stage 3 or 4 endometriosis, comparing ectopic to eutopic endometrium; their data showed a 3–9-fold increase in intraindividual expression of CYP1A1, a 5–53-fold intra-individual increase in gamma-SYN expression, and an elevation in Estrogen Receptor *β* (but not *α*). Additionally we have to consider that CYP1A1 and gamma-SYN are dioxin-inducible genes, and the observed upregulation of them could support, at least in part, the involvement of endocrine-disrupting agents in the pathogenesis of the disease. This is compatible with the results of Wu et al. [55], suggesting that polymorphisms of dioxin receptor complex components and detoxification-related genes jointly confer susceptibility to advanced-stage endometriosis. Considering the importance of gene-environment interactions, previously underlined, the Italian group of Vichi et al. [56] suggests that glutathione transferase (GST) gene polymorphisms per se do not increase the risk of developing endometriosis, although some genetic variants could modulate in different way the effect of endocrine-disrupting polychlorinated biphenyls (PCBs) implicated in the pathogenesis of the disease. Others [57], moreover, reported a linkage peak for endometriosis in rs11592737 SNP in the cytochrome P450 subfamily C (CYP2C19). Additionally, there is no evidence that CYP17 gene and Estrogen Receptor *α* gene could be considered as genetic risk factors for endometriosis, at least in Chinese women [163]. Furthermore, there is evidence of a significant correlation between polymorphism of the progesterone receptor gene (PROGINS) and endometriosis [58]. Focusing on E-cadherin, Govatati et al. [59] found increased membranous form expression of this protein in endometriosis in respect to controls, and moreover that the expression seems to be genotype dependent, according to various SNPs. Guo [60] on the contrary, reviewing 12 association studies on 5 genes (CYP17, CYP19, Androgen Receptor, Progesterone Receptor, and Estrogen Receptor) found no functional data that support a putative relationship of these genetic polymorphisms with endometriosis. Kim et al. [61] reported of a positive association between endometriosis and polymorphisms in insulin-like growth factors receptor genes: in fact, they found that women with endometriosis were observed 1.99 times more frequently to have IGF-II 820G>A SNP. A more recent study [62] of the same authors concluded that also polymorphisms in the insulin-like growth factor binding protein type 3 (IGFBP3) gene may be associated with advanced endometriosis. About this, others [63] suggest that the insulin receptor substrate (IRS)-2 G1057D polymorphism may be associated with an increased risk for endometriosis. Moreover, probably endometriosis pathogenesis may depend, at least in part, by mutation in autoimmunity genes. For example, Ammendola et al. [64] investigated on PTPN22, one of the few known shared-autoimmunity genes, and found that women carriers of the PTPN22(∗)T variant are significantly more susceptible to endometriosis than controls. Among the huge number of interleukin polymorphism, a case-control study based on Korean population [65] reported that the C627T polymorphism of the IL-2R beta gene may not be associated with the risk of endometriosis. Another study provided by Gonçalves-Filho et al. [66] Proposed that plasminogen activator inhibitor-1 4G/5G polymorphism may be associated with a risk of endometriosis-associated infertility. About the endometriosis-associated infertility André et al. [67] reported that FoxP3 polymorphisms can be associated with risk of idiopathic infertility (rs2280883 and rs2232368) and endometriosis (rs3761549), and this is congruent with other reports (see before) about the importance of FoxP3^+^ CD4^+^ Tregs in the pathogenesis of the disease. A more recent study [68] by the same group adds the hypothesis that FoxP3 and FCRL3 polymorphisms may have a cumulative effect in increasing the risk of developing endometriosis. Furthermore, Ruiz et al.'s results [69] demonstrated statistically significant differences in genetic variants in lysyl oxidase-like protein 4 (LOXL4) and complement component 3 (C3) in patients with endometriosis-associated infertility versus controls, and in patients with endometriosis versus controls, respectively. Medeiros et al. [70] found HMGA1 and HMGA2 gene rearrangements in the stromal component but not in the glandular component in 3 cases of polypoid endometriosis, suggesting a possible role of them in the pathogenesis of the disease. Despite the great number of work in the literature that evidenced the possible role of certain genetic variants in endometriosis susceptibility, the data are not conclusive. This consideration pushed some authors to try to resolve the dilemma, through a genome-wide association meta-analysis [71] showing three well-identified genes, which, if mutated by SNPs, seems to be associated with endometriosis: WNT4 encodes for wingless-type MMTV integration site family, member 4, and is important for the development of the female reproductive tract [164] and steroidogenesis [165]; VEZT encodes vezatin, an adherens junction transmembrane protein that is downregulated in gastric cancer [166]; GREB1 encodes growth regulation by estrogen in breast cancer 1, an early response gene in the estrogen regulation pathway involved in hormone dependent breast cancer cell growth [167]. For an accurate overview of the genetic variants reported in this chapter, refer to Table 1.

## 4. Organogenesis of the Müllerian Reproductive Tract

Embryogenesis phase, from 29th to 56th of development day, is defined as organogenesis, because during this period organs start to develop. The defects acquired during the organogenesis are usually more circumscribed than those of the first 28 development days (blastogenesis phase) and generally affect a single organ without compromising the survival of a developing organism. Having stated that, it seems to be essential to consider the embryological origin of various elements of genitourinary system in order to understand the pathogenesis of reproductive organs' diseases [168]. Around the 5th week of pregnancy Müllerian ducts (or paramesonephric ducts) appear as developing structures, and each part of them has a different developing pattern to form the final shape and function of the Müllerian-derived organs. The caudal extremity of the ducts is destined to merge and to constitute superior 2/3 parts of vagina and uterine cervix, the intermediate part fuses and creates uterine body, while the upper portions maintain their own independence and, opening in the coelomatic cavity (future peritoneal cavity), make fallopian tubes. In the same period, the renal system develops through the growth of urethral sketch, derived from Wolff's ducts (mesonephric ducts) within the mesenchyme of the metanephros. In similar times, the migration of the primordial germinal cells from the yolk sac leads to the formation of ovaries which arise from mesenchyme and from the epithelium of genital crest of the intermediate mesoderm, with female reproductive tract organogenetic processes different from those of mesonephros; therefore, the anomalies of Müllerian ducts are not associated, generally, with anomalies of ovary development [169].

## 5. Role of Hoxa Genes

It is widely accepted that during embryogenesis a key role is played by Hox (homeobox) genes: like is well reported by Zanatta et al. [170] in humans and mice, there are at least 39 Hox/HOX genes distributed in four groups lettered A, B, C, and D. These groups each comprise 9–13 genes and are distributed in the human chromosomes 7, 17, 12, and 2, respectively [171]. Two weeks after birth, a period that corresponds to the peak of the differentiation process in mice, Hoxa9, Hoxa10, Hoxa11, and Hoxa13 develop their characteristic spatial distribution throughout the Müllerian ducts: according to Taylor et al. [172] Hoxa-9 expression is limited to the fallopian tube; Hoxa-10 is expressed in the uterine epithelium, stroma, and muscle; Hoxa-11 is expressed in the cervical glands and epithelium (although it is also expressed in the uterine corpus); and Hoxa-13 is strongly expressed in the vaginal epithelium. The importance of the development and patterning of the uterus during embryogenesis is also supported by others [173]. Concerning these data, Hox genes are strictly involved in the differentiation of the paramesonephric duct into the mature female reproductive system, and moreover their persistent expression in the adult, as reported by Taylor et al. [172], may play a role in maintaining the developmental plasticity that is characteristic of this organ system. The late differential Hox axis formation may reflect the late differentiation of this organ system, and alterations in its expression could provoke, consequently, reproductive tract anatomic and functional anomalies. For example, it is documented in the mouse model that the losses of Hoxa-10 function provoke uterine alterations in decidualization and implantation phases, resulting in female infertility [173]; the mechanism by which Hoxa-10 altered expression causes these events is still unknown. Lim et al. [174] reported that in the absence of Hoxa-10, two prostaglandin E2 (PG)-E2 receptor subtypes, named EP3 and EP4, are inappropriately regulated by progesterone (P4). Moreover, they suggest that Hoxa-10 specifically mediates progesterone regulation of EP3 and EP4 in the uterine stroma, while epithelial cell functions seem to be not impaired. Regarding endometriosis, Painter et al. [175] conducted a rigorous genome-wide association study in 3,194 individuals with surgically confirmed endometriosis case and 7,060 controls from Australia and the UK. They found strong association signal located at 7p15.2 region, upstream of the plausible candidate genes NFE2L3 and HOXA-10. Others [176] reported that ectopic expression of Hoxa-9 in tumorigenic mouse ovarian surface epithelium cells gave rise to papillary tumors. In contrast, Hoxa-10 and Hoxa-11 induced morphogenesis of endometrioid-like and mucinous-like epithelial ovarian cancers, respectively. For this reason, it is hypothesized that inappropriate activation of a molecular program that controls patterning of the reproductive tract could address the development of different Müllerian-like features. Moreover, endometriosis progression could be caused, at least in part, by systematic repression of the genes involved in cell cycle and a specific regulation of the HOX genes [177]. Another of the most studied genes is EMX2: this is a fundamental transcription factor necessary for reproductive tract development, and its expression is strictly related to hormonal levels. About this, Daftary and Taylor [178] characterized menstrual cycle-dependent expression of EMX2 in endometrium from women with and without endometriosis, and they found that in endometriosis-free women EMX2 mRNA levels declined 50% in peri-implantation endometrium compared with levels in the proliferative phase. Moreover, they reported that sex steroids seem to regulate endometrial HOXA10 gene expression, which in turn negatively regulates EMX2. Conversely, others [179] identified a region of significant linkage peak for endometriosis on chromosome 7. For this reason, they screened coding regions and parts of the regulatory regions of three candidate genes with a known role in endometrial development and function, INHBA, SFRP4, and HOXA10, located under or very near this linkage peak: their results, surprisingly, indicated that the coding regions of these three genes do not harbor mutations responsible for linkage to endometriosis in the study population. Given all this, we have to take into account that endometriosis could arise, at least in part, by congenital abnormalities of the Hoxa genes during the embryonic life.

## 6. Role of Wnt Genes

Another well-known family of genes that influence remarkably the organogenesis of the Müllerian reproductive tract is Wnt (wingless-type MMTV integration site family). In particular, Wnt4 gene produces a secreted protein that suppresses male sexual differentiation, probably repressing the biosynthesis of gonadal androgen in female subjects. Moreover, like is reported by Biason-Lauber et al. [180] Wnt4 loss-of-function mutation could result in altered development of Müllerian-derived structures, like for example, phenotype resembling the Mayer-Rokitansky-Küster-Hauser syndrome, and to androgen excess. The limiting factor of these results is that they are derived from a small cohort [181]. Basing on a mouse model, it is demonstrated that Wnt genes expression is temporal and spatial differentiated according to phases of endometrial modification that occurs in pregnancy, concurrently with another gene family of Fzd (frizzled family receptor). About this, Hayashi et al., in a well-designed study [182], reported that during peri-implantation Wnt7a, Wnt7b, and Wnt11 mRNAs were abundantly detected in the endometrial epithelia. Conversely, Wnt16 mRNA was localized to the stroma surrounding the luminal epithelium (LE) on Gestational Day 4 and remained in the stroma adjacent to the LE but not in areas undergoing the decidual reaction. Moreover, they reported that this genes regulation seems to be addressed by ovarian steroid hormones, such as progesterone stimulated Wnt11 and estrogen stimulated Wnt4 and Wnt7b mRNA production. Same opinions are shared by Sonderegger's group [183], who moreover have showed that the canonical Wnt pathway seems to be involved in nuclear recruitment of *β*-catenin and activation of Wnt-dependent transcription factors. These transcription factors are critically involved in development and differentiation of the diverse reproductive tissues. Furthermore, they suggest that failures in Wnt signalling are associated with infertility, endometriosis, endometrial cancer, and gestational diseases such as complete mole placentae and choriocarcinomas. Others [184] found that E-cadherin, total *β*-catenin, and dephosphorylated *β*-catenin protein expressions were significantly higher in the mid-secretory endometrium of infertile patients with endometriosis or unexplained infertility compared to both luminal and glandular epithelial endometrium of healthy fertile controls. These alterations of the physiologic expression profile of the endometrium may underlie, at least in part, the pathogenesis of the endometriosis-related infertility.

## 7. Hoxa and Wnt Dynamic Interplay

**Figure 1:**
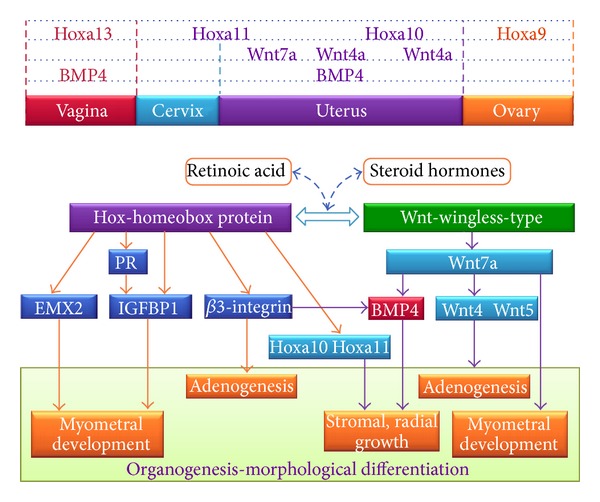
Hoxa and Wnt interplay mechanism. Hox—homeobox protein: this gene is part of the A cluster on chromosome 7 and encodes a DNA-binding transcription factor that may regulate gene expression, morphogenesis, and differentiation. More specifically, it may function in fertility, embryo viability, and regulation of hematopoietic lineage commitment. Wnt—wingless-type: this gene is involved in the development of the anterior-posterior axis in the female reproductive tract and also plays a critical role in uterine smooth muscle pattering and maintenance of adult uterine function. BMP—bone morphogenetic protein: the protein encoded by this gene is a member of the bone morphogenetic protein family which is part of the transforming growth factor-beta superfamily. The superfamily includes large families of growth and differentiation factors. Please refer to chapters 5 (Role of Hoxa genes), 6 (Role of Wnt genes), and 7 (Hoxa and Wnt dynamic interplay) for extensive explanation.

Like reported before, Hoxa and Wnt regulation and cell signaling pathways are utilized to pattern organs and to specify the fate of organogenesis. For this reason, it is widely accepted that the complex and accurate process of organogenesis of the Müllerian reproductive tract could be addressed, at least in part, by a strict interaction between these two gene families. This interaction is variable in the course of time according to the different phases of development, during which Hox transcription factors specify positional identity, and Wnt signaling provides spatial information and promotes asymmetric cell division [185]. Another evidence of these two gene families interplay was derived by the work of Klapholz-Brown et al. [186]. They analyzed the Wnt transcriptional effects on the stromal cells that make up the scaffold and infrastructure of epithelial tissues during key-event as development, regulation of stem cell self-renewal and differentiation, cell polarity, and morphogenesis. They confirmed the previous results and suggest that Wnt induced key transcription factors for the development, including Hox genes. Moreover, they verified that Wnt3a induced a gene, named GREMLIN2, which encodes a secreted bone morphogenetic protein (BMP) antagonist. Additionally, Wnt3a signals for the maintenance of stem cell niches, by inhibiting their differentiation and promoting their expansion in microenvironment, through the induction of high level of BMP antagonist production by nearby fibroblasts. The importance of Hox gene cluster in the correct development process is also shared by Deschamps [187], who underlined the well-conserved genetic pathways during evolution of different animal species. As suggested, Hox cluster regulation prefigured the temporal colinearity of expression of these genes in vertebrates, addressing the development of specific embryonic structures. Consequently, it is possible that any disturbance during the Müllerian reproductive tract development may lead, first of all, to altered molecular interactions, then to modified intercellular communications, and finally to readdressing of developing structures and coregulation of common downstream targets. Remembering that the form is the expression of the function, also the function of a temporally and spatially altered developing tissue will be compromised. For an accurate overview of the Hoxa and Wnt interplay mechanism reported in this chapter, refer to Figure 1.

## 8. The Importance of Endometrial Stem Cells

Recently, many studies in the literature support the hypothesis of the presence of adult stem cells in developed human body, within spatially selected areas named “niches” in which the surrounding microenvironment prevents the differentiation of this kind of cells. The uterus, in particular, seems to have a remarkable regenerative ability responsible for cyclical regeneration and remodelling throughout the reproductive life, responding to hormonal influence. One of the best example of this plasticity is represented by endometrial regeneration from the basal layer, which is fundamental for the replacement of the functionalis layer followed by its slough off during menses and parturition. Moreover, Cervelló et al. [188] isolated, identified, and characterized the side population (SP) cells corresponding to the human stromal and epithelial compartments and demonstrated that they display genotypic, phenotypic, and functional features of classic endometrial somatic stem cells (SSCs) population. This hypothesis is also shared by Tsuji's group [189], who showed that BCRP1/ABCG2, known as a marker of side population cells, was strongly expressed in the vascular endothelium and the epithelium of the basal layer of the endometrium. Furthermore, others [190], basing on a mouse model, suggest the importance of CD45-positive hematopoietic progenitor cells in regenerating the uterine epithelium. Others [191], moreover, reported that endometrial SP cells exhibit preferential expression of several endothelial cell markers compared to endometrial main population (EMP) cells. Additionally, they demonstrated that a medium specific for endothelial cell culture enabled endometrial SP cells to proliferate and differentiate into various types of endometrial cells, including glandular epithelial, stromal, and endothelial cells in vitro, whereas in the same medium, EMP cells differentiated only into stromal cells. Finally, their result concludes that endometrial SP could have the possibility, more than EMP, to direct in vivo angiogenesis and endometrial cell regeneration: this is consistent with the fact that endometriotic foci show a very high potential of angiogenic factor, related and needed for the worsening of the disease. Another very important study [192] provided evidence that human embryonic stem cells (hESCs) can be differentiated into cells with a human female reproductive tract epithelial cell phenotype. These findings support the hypothesis for which stem/progenitor cells, responsible for tissue regeneration and proliferative disorders of human endometrium, may be derived from Müllerian duct. The idea of the presence of adult stem cells in endometrial tissue pushed Schwab et al. [193] to make clonal analysis of human endometrial epithelial and stromal cells: their results demonstrated that inactive endometrium contains clonogenic epithelial and stromal cells, although this clonogenicity does not vary from the proliferative to secretory stage of the menstrual cycle, or between active cycling and inactive endometrium for both epithelial and stromal cells. Another astonishing results derived from Ikoma et al.'s study [194]: they searched for the presence of donor-derived cells in endometrium from patients who received bone marrow transplantation from male donors. Surprisingly, these donor-derived cells are capable of composing endometrium in recipients, remarking plasticity of bone marrow stem cells as well as a potential origin of endometrial stem cells. Though the reader may think that these results may arise from an isolated study, others confirm these findings [195] or share the same hypothesis [196]. According to Maruyama et al. [197], we think that this endometrial plasticity could derive, at least in part, by the presence of adult stem cell niches in the basal layer of endometrium. For this reason, there is not only the possibility that stem cell activities could play a role in the physiological remodelling and regeneration of the human uterus, but, moreover, that also the pathogenesis of gynaecological diseases such as endometriosis may be linked to stem cell genetic dysregulation, altered cell-to-cell communication, and, finally, misplacement of them.

## 9. Theories on Aetiopathogenesis of Endometriosis

Pathogenesis of endometriosis still remains controversial: immune, hormonal, genetic, and environmental factors seem to be involved. There are several theories that have been proposed to explain the pathogenesis of endometriosis. According to the Sampson's implantation theory [198], for example, during retrograde menstruation, eutopic endometrial cells reflux throughout the tubes to the peritoneal cavity, adhere to the peritoneal wall, proliferate and form endometriotic lesions, thereby triggering and advancing the disease [2, 3, 199] by the fact that the retrograde transport of endometrial cells was actually shown, as well as by the fact that the sites of greater frequency of the disease are fallopian tubes, ovaries, and pouch of the Douglas, those most easily reached by the refluxed cells. Moreover, there is evidence that nulliparous women and women with heavy and short menses are at higher risk of developing endometriosis [200]: experimental implantation of endometrial debris in peritoneum provokes growth of endometriotic foci in the animal model and an association between obstructed menstrual outflow and endometriosis [13]. However, this phenomenon could be observed in 90% of endometriosis-free women in reproductive age with pervious fallopian tubes and contrast with the relative low incidence of the disease [201]. Another hypothesis speculates that the endometriotic foci are derived by endometrial cells that enter in the uterine venous circulation and in this way could reach distant sites of implantation, like for example, brain [202], nasal mucosa [203], or spinal intradural [204]. This theory could explain distant sites of endometriosis, but we have to consider that the venous drainage of the uterus arrives to the lungs before to become oxygenated and pass in the arterial circulation: for this reason, according to this theory, we will have to find several cases of lung endometriosis which is not reflected in clinical facts. Another hypothesized way of dissemination of the endometrial cells is the lymphatic: there are in the literature several reports of endometriotic foci in the lymphatic node, but we think that this is not enough to support this theory. It is widely accepted also that endometrial cells could implant and proliferate in surgical scars after caesarean section or laparotomic/laparoscopic surgery [205] or in the episiotomy scars, and moreover the rupture of an endometrioma with leakage of its contents in the course of laparoscopic surgery or laparotomy could provoke dissemination in the peritoneal cavity. According to another theory endometriotic foci derive from peritoneal mesothelial cells of coelomic origin that undergo metaplasia transforming into endometrial cells. This hypothesis would explain the exceptional formation of foci in the bladder and prostate of males. Others, on the contrary, suggest that endometriosis could derive from a displacement of the primitive tissue that gives rise to endometrial cells, caused by incorrect reproductive tract organogenesis (embryonic derivation theory) [172, 206–208]. Other studies postulate that endometriotic cells may derive from “committed” stem cells that under the influence of different factors in the milieu evolve to form foci [191, 196]. Last but not least, basing on animal and experimental investigations, some authors suggest that in the pathogenesis of endometriosis play a role the exposure to environmental toxicants, such as dioxin and dioxin-like, although the mechanism(s) underlying this potential association are poorly understood [201]. Regardless of the correct etiopathogenetic theory, the implant and the proliferation of endometriotic cells seem to depend strictly on the local immune alterations present in the PF and inside of endometriotic cysts.

## 10. Discussion

As reported before, the elaborate process of female reproductive tract organogenesis is under the control of a wide range of genetic clusters that address spatially and temporally tissue-specific development of the pelvic structures. Among this genetic regulation, a key role is played by Hoxa and Wnt clusters, because they lead the transcription of different genes according to the different phases of the organogenesis, addressing correctly cell-to-cell interactions, allowing, finally, the physiologic morphogenesis. Hoxa and Wnt are important also later in life, because they seem to sustain endometrial plasticity according to hormonal influences and moreover preserve adult stem cells in the endometrial niches. Accumulating evidence [197] is suggesting that this particular type of stem cells is essential for the correct cyclic process of endometrial self-renewal after physiologic menses. Additionally, it was demonstrated that also other sources of stem cells, as for example, bone marrow derived pool [194], could repopulate endometrial niches under proper influences, modifying their genetic expression profile and consequently their cell phenotype and functions. Taking all this, there is the possibility that during organogenesis of Müllerian structures, Hoxa and Wnt alterations could provoke a disturbed development of female reproductive tract system, and for this reason primordial endometrial stem cells may be dislocated and disseminated in ectopic regions. These primordial endometrial stem cells, isolated or more probably organized in foci, could remain misplaced and quiescent until insults of various kinds (physical, chemical, hormonal) provoke expression of silent genes up to that moment, modifying in this way their phenotype, biological functions, and finally also immunogenicity. The discovery of endometriotic foci in fetal age [207, 208], outside the uterine cavity, supports the hypothesis that the disease originates during early organogenesis of the female reproductive tract, and develops in its clinical form in the postpubertal age as a result of hormonal influences. Moreover, Signorile et al.'s data [206] showed the ectopic presence of primitive endometrium, expressing both CA125 and oestrogen receptor, in 11% of female foetuses (4/36). Focusing on this evidence, we have to consider also that endometriosis affects approximately 10% of women in reproductive age [13, 14], and moreover that retrograde menstruation could be observed in 90% of endometriosis-free women in reproductive age with pervious fallopian tubes. Taking all together, all these pieces of evidence allow us to argue that the ectopic presence of primitive endometrium in female foetuses and the presence of endometriotic implants in women of reproductive ages are quite similar. Additionally, the locations of the ectopic primitive endometrium resembled the common locations for endometriosis in women [1, 3]. After certain stimuli, endometriotic cells could proliferate and form the classic ectopic foci and endometrioma, preferentially (but not only) in the peritoneal cavity. It is widely accepted that in the development of endometriosis, a key role is played by alteration in immune peritoneal microenvironment that may provoke failure of the peritoneal cavity scavenging mechanism in removing immunogenic endometriotic cells by macrophages. Like is reported before, modifications in the peritoneal microenvironment attract peripheral mononuclear cells, recruited from the blood into the peritoneal cavity, which secrete different pattern of cytokines, driving the following event of the disease. Once the endometriotic foci are established, in fact, the strict interaction between endometriotic and immune cells addresses toward a prevalence of Th1 profile cytokines in the PF at minimal and mild stages, whereas Th2 profile cytokines prevailed in severe stages [31]. Moreover, in the progression of the disease a key role could be played by impaired ratio of Th17 and Tregs populations [78–82].

## 11. Conclusion

Dysregulation of Wnt and/or Hox genes may affect cell migration during organogenesis and differentiation of Müllerian structures of the female reproductive tract, with possible dislocation and dissemination of primordial endometrial stem cells in ectopic regions, which have high plasticity to differentiation. We hypothesize that during postpubertal age, under the influence of different stimuli, these misplaced and quiescent endometriotic cells could acquire new phenotype, biological functions, and immunogenicity. So, these kinds of cells may differentiate, specializing in epithelium, glands, and stroma to form a functional ectopic endometrial tissue. This may provoke a breakdown in the peritoneal cavity homeostasis, with the consequent processes of immune alteration documented by peripheral mononuclear cells recruitment and secretion of inflammatory cytokines in early phases and of angiogenic and fibrogenic cytokines in the late stages of the disease.

## Figures and Tables

**Table 1:** Genetic variants associated with endometriosis.

Author(s)	Genetic variants
Treloar et al., 2005 [53]	Significant linkage on chromosome 10q26 and chromosome 20p13

Singh et al., 2008 [54]	Upregulation of dioxin-inducible CYP1A1 and gamma-SYN and of Estrogen Receptor *β*

Wu et al., 2012 [55]	Polymorphisms of dioxin receptor complex components and detoxification-related genes

Vichi et al., 2012 [56]	GSTP1(Ile/Ile) and GSTM1 null genotypes, modulating the effect of PCB153, PCB180, and of total PCBs

Painter et al., 2011 [57]	rs11592737 in the cytochrome P450 subfamily C (CYP2C19)

Costa et al., 2011 [58]	PROGINS polymorphisms (A1/A1, A1/A2 and A2/A2)

Govatati et al., 2012 [59]	E-cadherin −347GA/GA and −160A/A genotypes and −347GA/−160A/+54C and −347G/−160A/+54C haplotypes

Guo, 2006 [60]	CYP17, CYP19, Androgen Receptor, Progesterone Receptor, and Estrogen Receptor genetic variants are not associated with endometriosis

Kim et al., 2011 [61]	IGF-II 820G>A polymorphism

Kim et al., 2012 [62]	AAG haplotype allele of the −672A>G, −202A>C and c.95C>G polymorphisms in the IGFBP3

Çayan et al., 2010 [63]	IRS2 G1057D polymorphism

Ammendola et al., 2008 [64]	PTPN22(∗)T variant

Lee et al., 2009 [65]	C627T polymorphism of the IL-2R beta

Gonçalves-Filho et al., 2011 [66]	PAI-1 4G/5G polymorphism

André et al., 2011 [67]	FOXP3 polymorphisms (rs3761549)

Barbosa et al., 2012 [68]	Allele FOXP3 T for genotypes FCRL3TT/FOXP3CT, FCRL3CT/FOXP3CT, FCRL3CC/FOXP3CT

Ruiz et al., 2011 [69]	Variants in LOXL4 and complement C3

Medeiros et al., 2012 [70]	HMGA rearrangements

Nyholt et al., 2012 [71]	WNT4, VEZT, GREB1 polymorphism
